# Role of Adenylyl Cyclase Type 7 in Functions of BV-2 Microglia

**DOI:** 10.3390/ijms24010347

**Published:** 2022-12-25

**Authors:** Yawen Hu, Rebecca A. Hill, Masami Yoshimura

**Affiliations:** 1 Department of Comparative Biomedical Sciences, School of Veterinary Medicine Louisiana State University, Baton Rouge, LA 70803, USA; 2 Department of Microbiology, Immunology and Parasitology, Louisiana State University Health Sciences Center, New Orleans, LA 70112, USA

**Keywords:** microglia, adenylyl cyclase type 7, CRISPR-Cas9 gene editing, macrophage activation, alcohol

## Abstract

To assess the role of adenylyl cyclase type 7 (AC7) in microglia’s immune function, we generated AC7 gene knockout (AC7 KO) clones from a mouse microglial cell line, BV-2, using the CRISPR-Cas9 gene editing system. The ability of BV-2 cells to generate cAMP and their innate immune functions were examined in the presence or absence of ethanol. The parental BV-2 cells showed robust cAMP production when stimulated with prostaglandin-E_1_ (PGE_1_) and ethanol increased cAMP production in a dose-dependent manner. AC7 KO clones of BV-2 cells showed diminished and ethanol-insensitive cAMP production. The phagocytic activity of the parental BV-2 cells was inhibited in the presence of PGE_1_; AC7 KO BV-2 cells showed lower and PGE_1_-insensitive phagocytic activity. Innate immune activities of the parental BV-2 cells, including bacterial killing, nitric oxide synthesis, and expression of arginase 1 and interleukin 10 were activated as expected with small effects of ethanol. However, the innate immune activities of AC7 KO cells were either drastically diminished or not detected. The data presented suggest that AC7 has an important role in the innate immune functions of microglial cells. AC7’s involvement in ethanol’s effects on immune functions remains unclear. Further studies are needed.

## 1. Introduction

The microglial cell is a resident macrophage, comprising about 10–15% of the cells in the brain [1]. They are the first immune responders to infection and tissue injury in the central nervous system [2]. Like many macrophages, microglial cells are recruited to inflammatory regions by morphological activation, proliferation, and migration. Microglial cells are characterized as two distinct activated phenotypes: the classically activated (M1) phenotype associated with proinflammatory cytokines secretion and neurotoxic activity, and the alternative activated (M2) phenotype, which is associated with anti-inflammatory cytokine secretion and injury recovery [3,4]. These phenotypes show the complex roles of microglial cells in regulating inflammation and tissue homeostasis in the brain. Excessive alcohol consumption induces a higher incidence of health problems in many tissues/organs including the brain [4,5]. In the brain, alcohol abuse alters innate immune activation, which can cause brain atrophy, oxidative stress, myelin damage, neuroinflammation, and neuronal degradation [4,6,7]. Considering that microglial cells play a major role in innate immune responses in the brain, alcohol’s effects on the immune responses of microglia are of great interest.

cAMP is a well-known regulator of the innate immune functions of macrophages. cAMP inhibits phagocytic activities, bactericidal activity, and the production of proinflammatory mediators, and increases the production of anti-inflammatory mediators [8,9]. Similarly, cAMP’s regulatory role in the innate immune functions of microglia has been reported [10,11]. Adenylyl cyclase (AC) catalyzes the conversion of ATP to cAMP. There are ten known isoforms in the AC family: nine membrane-bound isoforms (AC1-AC9) and one soluble isoform (sAC). AC7 is a unique AC isoform activated by four different classes of heterotrimeric G proteins, including G_s_, G_i/o_, G_q_, and G_12/13_ [12,13], G_s_ via G_s**α**_, G_i/o_ via G_β**γ**_, G_q_ via G_q**α**_ through PKC, and G_12/13_ via G_12/13**α**_. Previous research shows AC7 plays an important regulatory role in the immune response of animals and that AC7 is highly expressed in immune cells including bone marrow derived macrophages (BMDM) [14]. To our knowledge, the role of a specific AC isoform in the immune responses of microglia is not known. Our research demonstrates that ethanol enhances AC activity in an isoform-specific manner; of these isoforms, AC7 is most enhanced by ethanol [15]. Thus, it is possible that ethanol affects immune functions via AC7. We hypothesize that AC7 is the major AC isoform that regulates the immune functions of microglia and that AC7 is a primary factor that controls ethanol’s effects on the immune responses of microglia.

In this study, we characterized the AC activity of BV-2 cells, a mouse microglial cell line [16]. We used prostaglandin E1 (PGE_1_) to activate all isoforms of AC in the cells and employed thrombin, sphingosine-1-phosphate (S1P), and ethanol to selectively enhance AC7 activity. The results indicated that AC7 was expressed in BV-2 cells. We isolated AC7 gene knockout (AC7 KO) BV-2 cell clones using the CRISPR-Cas 9 gene editing system and examined their AC activity and immune functions.

## 2. Results

### 2.1. Cyclic AMP (cAMP) Signaling of Parental BV-2 Cells

**Figure 1:**
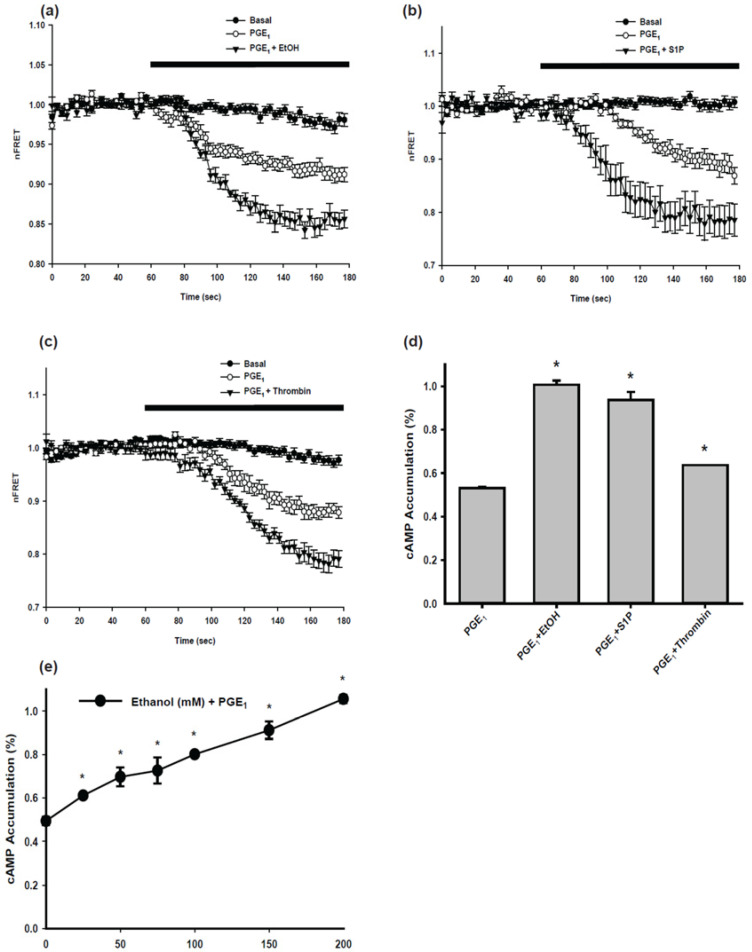
Characteristics of cAMP accumulation and Adenylyl cyclase (AC) activity in BV-2 mouse microglial cells. Temporal changes in intracellular cAMP concentration in response to 1 μM PGE_1_ and another stimulant, (**a**) 200 mM ethanol, (**b**) 100 nM S1P, or (**c**) 1 unit/mL thrombin, were monitored by FRET analysis detailed in the Section 4 Materials and Methods. Fluorescent images were captured every three seconds for three minutes. Normalized nFRET values were plotted over time. The horizontal bar above the graph indicates a 2 min period when indicated drugs were perfused. Data are represented as mean ± SEM (*n* = 8–20). Statistical analyses of the values from 100 s to 180 s were carried out by two-way repeated measures ANOVA. In all three experiments, values for Basal, PGE_1_, and PGE_1_ plus stimulant (ethanol, S1P, or thrombin) were statistically different from each other. AC activity of BV-2 mouse microglial cells. (**d**) AC activity of BV-2 cells in response to 1 min drug stimulation was measured using a cAMP accumulation assay. Cells were incubated with 500 μM IBMX for 10 min and stimulated with 1 μM PGE_1_ or PGE_1_ plus another stimulant: 200 mM ethanol, 100 nM S1P, or 1 unit/mL thrombin for 1 min. Data are represented as mean ± SEM in triplicate. * indicates values are significantly larger than the value for cells treated with PGE_1_ alone at *p* < 0.05 using one-way ANOVA. (**e**) The effects of ethanol on PGE_1_-stimulated AC activity were examined. Cells were incubated with 500 μM IBMX for 10 min and stimulated with 1 μM PGE_1_ with varying concentrations of ethanol (0, 25, 50, 75, 100, 150, or 200 mM). * Indicates value is significantly larger than PGE_1_ alone at *p* < 0.05 using one-way ANOVA.

Temporal changes in intracellular concentrations of cAMP were examined using a fluorescence resonance energy transfer (FRET) based cAMP sensor, Epac1-camps. Prostaglandin-E_1_ (PGE_1_) was utilized to stimulate cAMP production in Epac1-camps expressing BV-2 cells with or without ethanol, and AC7 specific activators, S1P, or thrombin. Stimulation with PGE_1_ led to a rapid decrease in nFRET; indicating an increase in cAMP production. Normalized nFRET values were an indicator of cAMP levels; these values were plotted over time (Figure 1). When PGE_1_ was combined with other stimulants (ethanol, S1P, or thrombin), nFRET was reduced further, indicating that a combination of PGE_1_ and ethanol, S1P, or thrombin increases intracellular cAMP more than PGE_1_ alone. We have been studying AC activity regulations over many years and found repeatedly that ethanol alone, S1P alone, and Thrombin alone do not increase cAMP [15,17,18]. Thus, these conditions were not tested. The results indicated that AC7 was a major AC isoform in BV-2 cells.

To examine the activity of adenylyl cyclase (AC), cAMP accumulation was measured in the presence of 3-isobutyl-1-methylxanthine (IBMX), a non-specific phosphodiesterase inhibitor. BV-2 cells under basal conditions did not show detectable AC activity. PGE_1_ induced AC activity in the cells stimulated; this activity was further enhanced in the presence of PGE_1_ in combination with ethanol, S1P, or thrombin (Figure 1d). Ethanol increased PGE_1_-induced AC activity in a concentration-dependent manner (Figure 1e). When 1µM PGE_1_ was combined with ethanol at a physiologically relevant concentration (25 mM), there was a significant increase in AC activity compared to PGE_1_ alone.

### 2.2. Isolation and Identification of AC7 KO Clones of BV-2 Cells

**Figure 2:**
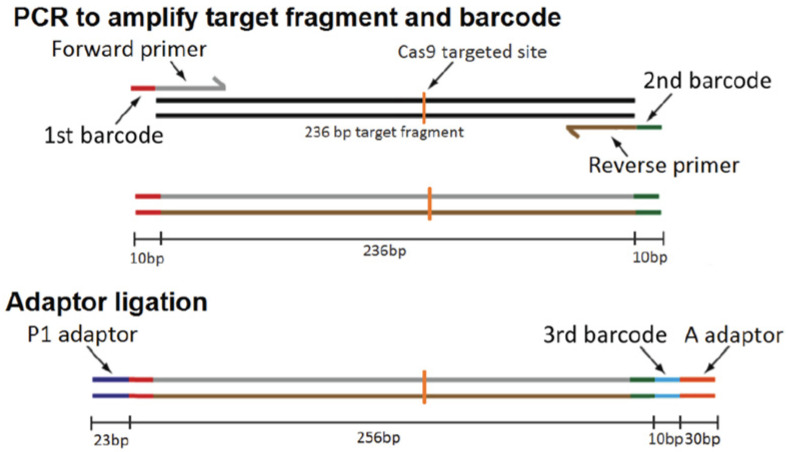
Preparation of NGS samples.

A surrogate reporter plasmid (pMRS) and a CRISPR-Cas9 expression plasmid (pX330) that carry an AC7 single-guide RNA (sgRNA) coding sequence were co-transfected into BV-2 cells. Cells expressing a high level of enhanced green fluorescent protein (eGFP) were sorted using FACS. The sorted cells were clonally cultivated to identify mutation resulting from non-homologous end joining repair events. Using a next-generation sequencing (NGS) strategy, individual cell clones harboring the mutation of interest were identified (Figure 2). 3922 eGFP-positive cells out of 20 million sorted cells were isolated by FACS. Of the 3922 eGFP-positive cells, 151 cells survived and formed colonies; these colonies were characterized using NGS. 9 out of 151 surviving clones were AC7 biallelic KO cells. 53 and 89 surviving clones were monoallelic AC7 KO clones and WT clones, respectively. We did not observe any changes in viability, growth rate, and cell morphology in AC7 KO clones compared to the parental cells. The mutations in the AC7 biallelic KO clones are shown in Table 1. Out of 9 AC7 KO clones, 6 of them showed one base deletion in both alleles. There is a minute possibility that one allele had a change (deletion or insertion) that escaped designed PCR amplification and was not detected. Three clones (#2, #4, and #6) have clear unmistakable mutations.

One of the WT clones and AC7 KO clones, No. 2 and No. 4, respectively, were chosen for further confirmation. The five most probable off-target sites were identified in genes named Etl4, Mrps28, Lrrc63, Epb41l1, and Prkca. Genomic DNA isolated from the parental cells and the three clones had the same WT sequences in these five loci. The authenticity of the three clones was examined by short tandem repeat (STR) marker profiling using eight STR markers and species-specific PCR evaluation. The results indicated the clones originated from the parental BV-2 cells; there was no interspecies contamination.

### 2.3. AC Activity of AC7 KO Clones of BV-2 Cells

**Figure 3:**
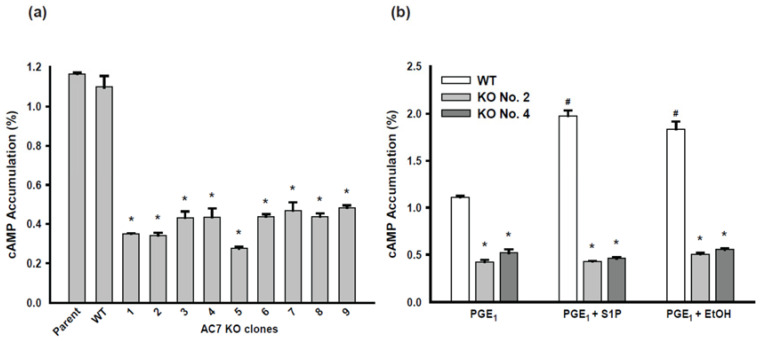
AC activity of AC7 knockout (KO) clones of BV-2 mouse microglial cells. (**a**) Cells were stimulated with 1 μM PGE_1_ for 1 min after 10 min of incubation with 500 μM IBMX. Data are represented as mean ± SEM in triplicate. The value for the wild-type clone (WT) does not differ from the value of the parental BV-2 cells. * indicates the values for AC7 KO clones No. 1 to No. 9 are significantly smaller than the value of the parental BV-2 cells at *p* < 0.05 using one-way ANOVA. (**b**) Cells of the WT clone and AC7 KO clones No. 2 and No. 4 were incubated with 500 μM IBMX for 10 min and stimulated with 1 μM PGE_1_ or PGE_1_ plus another stimulant: 100 nM S1P or 200 mM ethanol for 1 min. Data are represented as mean ± SEM in triplicate. * indicates the values of clones No. 2 and No. 4 are significantly smaller than the value of the WT clone at *p* < 0.05 using one-way ANOVA. # indicates that, for the WT clone, values for PGE_1_ plus S1P and PGE_1_ plus ethanol are significantly larger than the value for PGE_1_ alone at *p* < 0.05 using one-way ANOVA. There are no significant differences among the three treatment groups in the AC7 KO clone No. 2 and No. 4.

Using the cAMP accumulation assay, AC activity of one of the WT clones and the nine AC7 KO clones were compared with the AC activity of the parental BV-2 cells after stimulation with PGE_1_ (Figure 3a). The WT clone exhibited a comparable level of AC activity to the parental BV-2 cells, while all nine AC7 KO clones showed significantly smaller AC activities (approximately 35% of the parental cells). AC7 KO clones No. 2 and No. 4 were chosen for subsequent analysis. The PGE_1_-induced AC activity of the WT clone was significantly increased by the addition of S1P or ethanol (Figure 3b). This is similar to the observation with the parental BV-2 cells shown in Figure 1d. Both S1P and ethanol did not increase the PGE1-induced AC activity of AC7 KO clones No. 2 and No. 4.

### 2.4. Innate Immune Responses of AC7 KO Clones of BV-2 Cells

#### 2.4.1. Phagocytic Activity of BV-2 Cells

**Figure 4:**
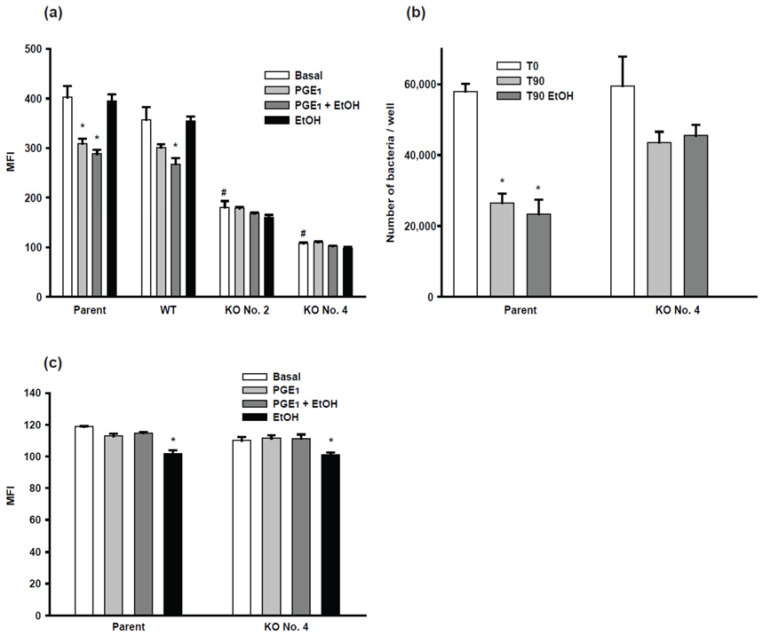
Effects of AC7 KO on phagocytosis, bacteria-killing, and oxidative burst of BV-2 cells. Assays are detailed in the Section 4 Materials and Methods. (**a**) Phagocytosis assay with parental cells, the WT clone, and two AC7 KO clones were carried out in the presence of no drugs (Basal), 10 μM PGE_1_, 10 μM PGE_1_ plus 50 mM ethanol, or 50 mM ethanol. Data are presented as mean fluorescent intensity (MFI) and are represented as mean ± SEM in triplicate. * indicates the value is significantly smaller than the corresponding basal value. # indicates the value is significantly smaller than the basal value of parental cells at *p* < 0.05 using one-way ANOVA. (**b**) Bacteria-killing activity of the parental cells and one of the AC7 clones was measured by counting live bacteria before (T0) and after 90 min incubation (T90). One set of samples included 50 mM ethanol during the incubation (T90 EtOH). Data are represented as mean ± SEM in triplicate. * indicates the value is significantly smaller than the corresponding T0 value at *p* < 0.05 using one-way ANOVA. (**c**) Oxidative burst was measured under the basal condition, 10 μM PGE_1_, 10 μM PGE_1_ plus 50 mM ethanol, or 50 mM ethanol. Data are presented as MFI and are represented as mean ± SEM in triplicate. * indicates the value is significantly smaller than the corresponding basal value.

cAMP signaling has been known to regulate the phagocytosis of macrophages [8,9]. To investigate whether ethanol-enhanced cAMP affects phagocytosis of BV-2 cells and whether AC7 KO affects phagocytosis of BV-2 cells, we tested the phagocytic activity of both WT and AC7KO BV-2 clones compared to the parental BV-2 cells (Figure 4a). Under the basal condition, the phagocytosis rate of the WT clone was similar to the parental cells, while AC7 clones No. 2 and No. 4 showed significantly reduced phagocytic activity. The phagocytic activity of the parental cells was reduced in the presence of PGE_1_. Ethanol, alone or in combination with PGE_1_, did not result in significant effects on the parental cell’s phagocytosis rate. The WT clone’s profile was similar to the parental cells. PGE_1_ stimulation with or without ethanol did not have any significant effects on the phagocytosis rate of the AC7 KO clones.

#### 2.4.2. Bacteria-Killing Activity of BV-2 Cells

As a resident macrophage, the bacteria-killing capability is an essential function of microglia. To investigate the bacteria-killing activity of BV-2 cells, we measured the numbers of live bacteria at two time points in the bacterial killing process. “Before killing” represents the number of bacteria when cells just phagocytosed bacteria. “After killing” represents the number of live bacteria 90 min after initiation of phagocytosis. Before killing, both parental cells and AC7 KO clone No. 4 showed a similar number of live bacteria. After killing, the parental cells showed a significantly reduced number of live bacteria (less than 50%), while AC7 KO clone No. 4 showed no significant reduction in the number of live bacteria (Figure 4b). The addition of ethanol did not alter the killing activity of the parental cells or the AC7 KO clone. These results suggest that removing AC7 expression compromised the bacteria-killing activity of BV-2 cells; under the conditions detailed here, ethanol did not affect the bacteria-killing activity of BV-2 cells.

#### 2.4.3. Oxidative Burst Activity of BV-2 Cells

Oxidative burst is another critical factor to evaluate macrophage’s immune function in response to bacterial infections. The oxidative burst activity of the parental cells was not affected by the addition of PGE_1_ or PGE_1_ plus ethanol but ethanol significantly reduced the oxidative burst activity. AC7 KO clone No. 4 showed very similar oxidative burst activity and ethanol effect on this activity as seen for the parental BV-2 cells (Figure 4c). Our results indicate that AC7 expression does not affect the oxidative burst activity of BV-2 cells; the effect of ethanol on this activity is independent of AC7.

#### 2.4.4. Classical Activation (M1) of BV-2 Cells

**Figure 5:**
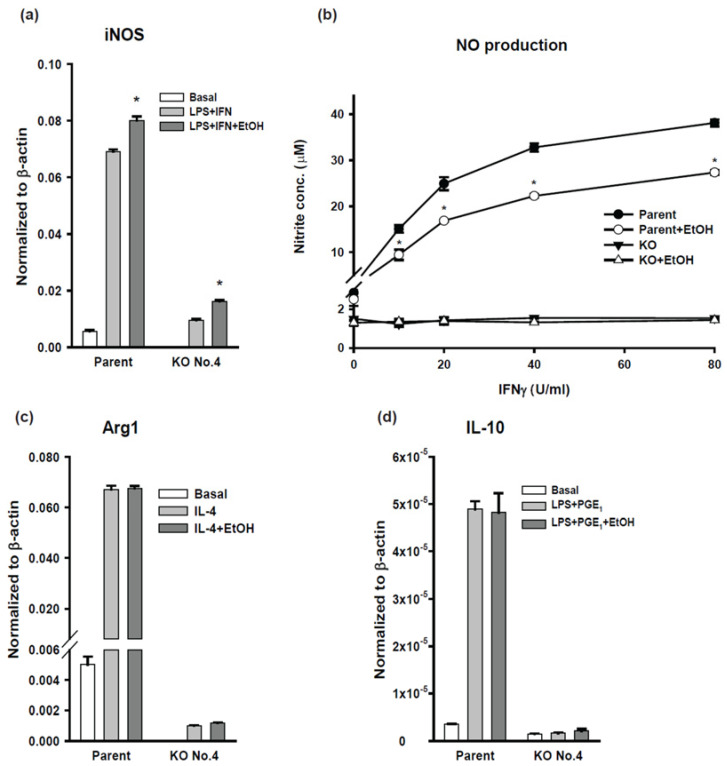
Activation of BV-2 AC7 KO cells. (**a**) The quantity of iNOS mRNA was measured by qRTPCR after activation with no drugs (Basal), 3 ng/mL lipopolysaccharide (LPS) plus 40 U/mL interferon (IFN)-γ or 3 ng/mL LPS plus 40 U/mL IFN-γ plus 50 mM ethanol as indicated. The expression level of mRNA was normalized to β-actin mRNA in the same sample. Data are represented as mean ± SEM in triplicate. The basal value for KO No. 4 is 4.8 × 10^−5^ ± 4.0 × 10^−6^. * indicates the value is significantly larger than the corresponding LPS + IFN value at *p* < 0.05 using one-way ANOVA. (**b**) NO production was assessed by measuring nitrite in the culture medium as described in the Section 4 Materials and Methods. Cells were incubated with 3 ng/mL LPS and indicated concentrations of IFN-γ with or without 50 mM ethanol. Data are represented as mean ± SEM in triplicate. * indicates that in the parental cells, IFN-γ concentration-dependently increased NO production and ethanol significantly decrease NO production at *p* < 0.05 using two-way ANOVA. For AC7 KO clone No. 4, IFN-γ or ethanol did not have any significant effects on NO production. (**c**) The quantity of Arg1 mRNA was measured by qRTPCR under basal conditions or activated with 20 U/mL interleukin 4 (IL-4) or 20 U/mL IL-4 plus 50 mM ethanol. The expression level of mRNA was normalized to β-actin mRNA in the same sample. Data are represented as mean ± SEM in triplicate. The basal value for KO No. 4 is 3.9 × 10^−6^ ± 6.0 × 10^−7^ (**d**) The quantity of interleukin 10 (IL-10) mRNA was measured by qRTPCR under basal conditions or activated with 100 ng/mL LPS plus 10 μM PGE_1_ or 100 ng/mL LPS plus 10 μM PGE_1_ plus 50 mM ethanol. The mRNA expression level was normalized to β-actin mRNA in the same sample. Data are represented as mean ± SEM in triplicate. The three values of AC7 KO cells are not significantly different from one another.

BV-2 cells can be differentiated into different phenotypes, including classical activation (M1), alternative activation (M2a), and regulatory (M2b) [19]. M1 phenotype of BV-2 cells was activated by interferon (IFN)-γ and lipopolysaccharide (LPS), and the representative marker is inducible nitric oxide synthase (iNOS) or its product, nitric oxide (NO). iNOS mRNA expression of the parental BV-2 cells was induced by LPS and IFN-γ. Expression was further increased with the addition of ethanol (Figure 5a). iNOS mRNA expression in AC7 KO clone No. 4 was similarly induced by LPS and IFN-γ and was further increased with the addition of ethanol; however, the amount of the iNOS mRNA was greatly diminished compared to that of the parental cells. NO production was assessed by measuring nitrite in the culture medium (Figure 5b). In the presence of LPS, nitrite concentration in the medium in which the parental cells were cultured increased in an IFN-γ concentration-dependent manner. The addition of ethanol significantly decreased nitrite production and there was no detectable increase in nitrite production by AC7 KO clone No. 4 in response to IFN-γ addition. Our results indicate that AC7 expression is critical for the expression of iNOS mRNA and the production of NO. Ethanol appeared to have opposite effects on iNOS mRNA expression and NO production.

#### 2.4.5. Alternative Activation (M2a) of BV-2 Cells

M2a phenotype of BV-2 cells was activated by interleukin 4 (IL-4) and the representative marker is arginase I. Arginase I mRNA expression was increased in parental BV-2 cells in response to IL-4 while AC7 KO clone No. 4 showed a very small increase in arginase I mRNA expression (Figure 5c). Ethanol had no significant effects on IL-4 induced arginase I mRNA expression in the parental cells or the AC7 KO clone. The results suggest that AC7 expression plays an important role in the induction of arginase I mRNA expression.

#### 2.4.6. Regulatory Activation (M2b) of BV-2 Cells

M2b phenotype of BV-2 cells was activated by LPS and PGE_1_ and the representative marker is interleukin 10 (IL-10). The expression of IL-10 mRNA was significantly increased in the parental BV-2 cells in response to the M2b activation, while the AC7 KO clone No. 4 did not show any significant change in IL-10 mRNA expression in response to the M2b activation (Figure 5d). Ethanol at 50 mM appeared to have no detectable effects on IL-10 mRNA expression in either the parental cells or the AC7 KO clone. The results suggest that under the condition used, AC7 expression is essential for the induction of IL-10 mRNA expression in BV-2 cells.

## 3. Discussion

Although cAMP is well-known as a regulator of the immune system, the regulatory role of different AC isoforms in the immune system is not fully understood. In this study, we examined the role of AC7 isoform and ethanol in the innate immune responses of BV-2 cells, a mouse microglial cell line. We have previously reported that alcohol enhanced the activity of AC in an isoform-specific manner [15]. AC7 is highly expressed in immune cells, including BV-2 cells [14,20]. Therefore, we expect that AC7 plays an important role in the immune response of BV-2 cells and that a portion of ethanol’s effects on the immune response is mediated by AC7.

The observed regulation of AC activity of the parental BV-2 cells suggests that AC7 is a predominant AC isoform expressed in BV-2 cells. PGE_1_, a commonly used activator of G_s_, increased the AC activity of the parental BV-2 cells; this was further enhanced by ethanol in a concentration-dependent manner. Ethanol at 25 mM significantly increased PGE_1_-stimulated AC activity of the parental BV-2 cells. This is similar to the effect of ethanol on the AC activity of the HEK 293 cells overexpressing AC7 [21]. The temporal change of intracellular cAMP of the parental BV-2 cells in response to PGE_1_ plus ethanol is similar to that of Hela cells overexpressing AC7 [22] and NIH 3T3 cells overexpressing AC7 [23]. S1P and thrombin enhanced G_s_-stimulated AC activity of the parental BV-2 cells. This is similar to the AC activity of RAW 264.7 cells, BMDM, and HEK 293 cells overexpressing AC7 [17,18]. The stimulatory effects of S1P and thrombin on AC activity are unique to AC7 and mediated by G_12/13_ [17,18]. Our observation of AC activity of the parental BV-2 cells is consistent with the notion that AC7 is the major AC isoform expressed in BV-2 cells and suggests that AC7 is the dominant AC isoform of microglia.

To confirm that AC7 is the major AC isoform expressed in BV-2 cells, we generated AC7KO BV-2 cells using CRISPR-Cas9 mediated genome editing. The process generated nine biallelic AC7 KO clones; further analysis of two of the clones, No. 2 and No. 4, indicated that they were authentic BV-2 AC7 KO clones. The AC7 KO clones of BV-2 cells had AC activity less than half of the parental BV-2 cells and did not respond to stimulation with S1P or ethanol. A previous study showed that BV-2 cells express AC3, AC4, and AC7. The level of AC7 mRNA was approximately twofold higher than that of AC3 or AC4 [20]. The AC activity data presented herein are consistent with the mRNA data previously published. It is unclear if the expression of other AC isoforms increased in AC7 KO cells to compensate for the loss of AC7. Nevertheless, the results indicate that AC7 is the major AC isoform expressed in BV-2 cells and suggest that AC7 is the major isoform expressed in microglia.

Increased cAMP has been shown to inhibit phagocytic activity of macrophages and microglia [8,9,24]. Therefore, we expected that AC7 KO BV-2 cells may have higher phagocytic activity or that AC7 KO BV-2 cells may not respond to stimulation of cAMP production. PGE_1_ inhibited the phagocytic activity of the parental cells and the WT clone (Figure 4a). As expected, PGE_1_ did not inhibit the phagocytic activity of the AC7 KO clones. However, the AC7 KO clones showed significantly lower phagocytic activity compared to the parental cells or the WT clone. This is unexpected and suggests that the expression of AC7 may be favorable for phagocytosis. Ballinger et al. showed that during phagocytosis, there were transient bursts of cAMP generation near phagosomes while total cAMP generation did not change [25]. This suggested that a compartmentalized increase in cAMP may be required for phagocytosis while bulk cAMP stayed constant. We speculate that AC7 may play a role in this. Ethanol alone had no significant effect on phagocytosis. Even a combination of ethanol and PGE_1_ had no effect on phagocytosis over PGE_1_ alone. Considering the effect of ethanol on cAMP production by BV-2 parental cells and the WT clone, this was unexpected. This could be because cAMP generated by PGE_1_ alone was enough for the inhibition of phagocytosis and no further inhibition was possible by additional cAMP. Ethanol’s effects on phagocytotic activity of microglia are diverse. Early reports using primary microglial cells in culture showed that the ethanol’s effect was stimulatory in one study [26], while it was inhibitory in another [27]. More recent studies also showed a similar disparity [28]. This complexity is reminiscent of ethanol’s effects on phagocytic activity of other types of macrophages [29]. The diversity of ethanol effects on phagocytosis of microglial cells appears to stem from the diversity of experimental conditions employed, such as concentration and duration of ethanol exposure and materials used of phagocytosis.

The parental cells showed bacteria killing activity (Figure 4b). However, AC7 KO clone No. 4 lost the killing ability under the condition used. This suggests that the expression of AC7 is necessary for the killing activity. The oxidative burst activity of the parental cells and AC7 KO clone No. 4 was similar; the difference in the oxidative burst between the two cell populations cannot explain the difference in killing activity between the parental cells and the AC7 KO clone.

We examined the ability of BV-2 cells to respond to activation signals by applying the protocols developed for macrophages [19]. The parental BV-2 cells responded to the activation protocols for M1, M2a, and M2b as expected [11] and expressed specific genes characteristic to the corresponding activation programs (Figure 5). Ethanol increased the expression of iNOS but decreased NO production. The inhibitory effect of ethanol on NO production by iNOS was described previously [26,30,31]. Our results are consistent with the previous reports. there is currently no consensus regarding the effect of ethanol on the expression of iNOS.

To our surprise, AC7 KO clone No. 4 showed either drastically reduced responses or no responses to the activation protocols for M1, M2a, and M2b. The M1 activation is a typical inflammatory induction and cAMP has been shown to inhibit this induction [9]. Therefore, we expected that iNOS expression and NO production by the AC7 KO cells would be higher than that of the parental cells. Using BMDM carrying either wild-type (WT) AC7 genes (+/+) or defective AC7 genes (−/−), Duan et al. showed that AC7 defective BMDM had higher TNFα expression than BMDM carrying WT genes in response to LPS treatment [14]. TNFα is another marker of inflammatory response induced by the M1 activation protocol. This discrepancy between the current study and the previous study could be due to the difference in the cell type employed: microglial cell line, BV-2 vs. BMDM, or the difference in examined gene: iNOS vs. TNFα, or the difference in the induction condition used: LPS plus IFN-γ vs. LPS alone. cAMP is a positive modulator for M2a and M2b activation [11], thus, we expected a reduction in the expression of Arg1 and IL-10 in the AC7 KO cells. We observed that the AC7 KO cells retained ~35 % of the original AC activity of the parental cells. Thus, almost complete loss of Arg1 and IL-10 expression in the AC7 KO cells is unexpected. The activation deficit of the AC7 KO cells suggests that AC7 is critically important for the microglia activation of M1, M2a, and M2b phenotypes. Considering that the AC7 KO cells retain some AC activity, complete inhibition of activation phenotypes in the AC7 KO cells could be due to changes/loss of cAMP in a specific intracellular compartment responsible for the activations.

It is not possible to eliminate the possibility of artifacts during the selection of AC7 KO clones. We confirmed the high likelihood of authenticity of the AC7 clones by examining the five most probable off-target sites and eight STR markers and evaluating the contamination of cells derived from other species. To validate the current findings regarding the activation deficits of the AC7 KO cells, pharmacological intervention (e.g., use of cAMP analogs) and/or reintroduction of AC7 expression could be employed to examine if these treatments can reverse the observed activation deficits. It is also possible that the BV-2 cell line may not retain the original characteristics of macroglia [32]. Further studies using primary microglia or live animals are needed.

In conclusion, we have generated permanent biallelic AC7 KO BV-2 cells and examined their capacity to generate cAMP and their immune functions. Their functional profiles were altered from parental BV-2 cells in an unexpected way. Our results demonstrate that as a dominant AC isoform in BV-2 cells, AC7 is a critical factor in regulating phagocytosis, bacterial killing, and microglia activation. It was unclear if AC7 plays a vital role in alcohol-related CNS diseases since the effects of AC7 KO were unexpectedly detrimental to immune functions.

## 4. Materials and Methods

### 4.1. Reagents

Ethanol and LPS from *E. coli* O55: B5 was bought from Sigma-Aldrich (St. Louis, MO, USA). Prostaglandin E_1_ (PGE_1_), 3-isobutyl-1-methylxanthine (IBMX), sphingosine-1-phosphate (S1P), and dihydrorhodamine 123 (DHR123) were bought from Caymal Chemical (Ann Arbor, MI, USA). [α-^32^P] ATP was bought from Perkin Elmer (Boston, MA, USA). [2,8-^3^H] adenine and [8-^14^C] cAMP were bought from Moravek Biochemicals (Brea, CA, USA). IFN-γ was bought from Abcam (Cambridge, MA, USA). IL-4 was purchased from R & D Systems (Minneapolis, MN, USA).

### 4.2. Cell Culture and Transfection

A BV-2 mouse microglial cell line [16], was purchased from Banca Biologica e Cell Factory (Genova, Italy). The cells were cultured in Dulbecco’s modified Eagle’s medium (Gibco, Billings, MT, USA), supplemented with 10% fetal bovine serum (Hyclone, Logan, UT, USA), streptomycin (50 μg/mL) and penicillin (50 μg/mL), in a humidified atmosphere of 95% air and 5% CO_2_ at 37 °C. BV-2 cells were transiently transfected with CalFectinTM Mammalian DNA Transfection Reagent (SignaGen, Rockville, MD, USA) in accordance with the manufacturer’s protocol. The efficiency of transfection was examined 24 to 48 h post transfection by visually observing the expression of fluorescent proteins.

### 4.3. Real-Time Assessment of Intracellular cAMP

cAMP measurement was conducted using fluorescent resonance energy transfer (FRET) as described previously [22]. Briefly, the parental BV-2 cells were transiently transfected with a plasmid that expresses Epac1-camps [33]. Fluorescence images of cells in a perfusion chamber were acquired by a fluorescent imaging workstation using SlideBook 5.1 software. FRET signals were analyzed by sensitized FRET measurement described previously [34]. nFRET values were calculated as described in [22] and normalized to the mean nFRET before the addition of drugs.

### 4.4. cAMP Accumulation Assay

The AC activity of parental BV-2 cells and AC7 KO clones were measured using the cAMP accumulation assay described previously [35]. The modification made for this study is the replacement of Dulbecco’s modified Eagle medium (0.5 mL per well) without phenol red with KRH buffer (129 mM NaCl, 1 mM CaCl_2_, 4.8 mM KCl, 5 mM NaHCO_3_, 1.2 mM MgCl_2_, 1.2 mM KH_2_PO_4_, 2.8 mM glucose, 10 mM HEPES, pH7.2). The drugs used for stimulation were indicated in the figure legends. The cAMP accumulation was calculated as a percentage of cellular pool of [H^3^]ATP converted to [H^3^]cAMP: cAMP accumulation (%) = [H^3^]cAMP/([H^3^]cAMP + [H^3^]ATP) × 100.

### 4.5. Isolation of Biallelic AC7 Knockout (KO) Clones of BV-2 Cells

#### Construction of CRISPR-Cas9 Screening Plasmids

pX330, a CRISPR-Cas9 expression plasmid [36] was obtained from Addgene (Watertown, MA, USA). pMRS, a surrogate reporter plasmid [37], was obtained from PNA Bio (Newbury Park, CA, USA). The sequence for single-guide RNA (sgRNA) was selected from the mouse AC7 genomic sequence using several CRISPR sgRNA design tools available online [38,39,40,41,42]. The single sequence corresponding to AC7 sgRNA (5′- TCTCCACACGAAGCTTCCGACGG -3′) was chosen and inserted into pX330 and pMRS using appropriate restriction sites of the plasmids. DNA sequencing confirmed the sequencing of the resulting plasmids. The gRNA sequence was selected from the C1 domain of the AC7 coding sequence that is important for the catalytic activity of the protein.

### 4.6. Isolation of BV-2 Cells That Exhibit CRISPR-Cas9 Mediated Mutation

**Figure 6:**
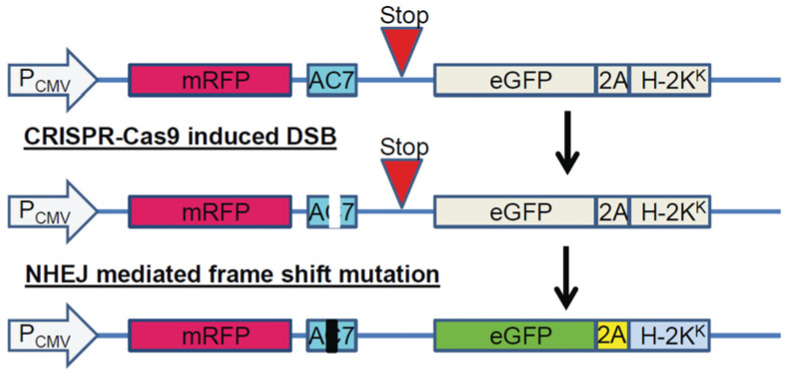
Surrogate Reporter. The figure shows how the reporter generates mRFP-eGFP fusion protein because of a gene-editing event. P_CMV_: cytomegalovirus promoter; mRFP: coding sequence of monomeric red fluorescent protein; AC7: AC7 gene derived target sequence; Stop: in-frame stop codon; eGFP: coding sequence of enhanced green fluorescent protein; 2A: self-cleaving peptide; H-2K^K^: mouse MHC class I alloantigen; DSB: double-strand break; NHEJ: non-homologous end-joining.

BV-2 cells were transiently transfected with pX330 and pMRS that carry AC7 sgRNA sequence. If CRISPR-Cas9 mediated mutation occurs, cells could express mRFP-eGFP fusion protein (Figure 6). Twenty-four hours after transfection, fluorescence-activated cell sorting (FACS) was utilized to isolate cells expressing enhanced green fluorescent protein (eGFP) and deposited individually into 96-well plates. Cells were cultured until a colony was formed.

### 4.7. Next-Generation Sequencing (NGS) Analysis of BV-2 Cell Clones

NGS analysis of BV-2 clones was carried out by a high-throughput strategy as described previously [43] using the barcode system of Ion Torrent. Briefly, the DNA of BV-2 cell clones was isolated by incubating cell pellets using 25 mM NaOH/0.2 mM EDTA solution at 95 °C for 2 h and further purified by DNA Clean & Concentrator kits (Zymo Research, Irvine, CA, USA) in accordance with the manufacturer’s instruction. The genomic DNA covering the targeted sequence (236 bp) was amplified by PCR using primers including barcodes (Xpress barcode 1 to 20) to identify 96 different clones. The PCR products from a single 96-well plate were pooled, and the Ion P1 adaptor and the barcoded Ion A adaptor were ligated to distinguish clones derived from different 96-well plates (Figure 2). NGS was carried out using the Ion PGM System.

### 4.8. Identification of BV-2 Cell Clones That Carry Biallelic AC7 KO Mutation

NGS data were analyzed using The Galaxy, online software kits [44]. All the sequences derived from a single clone were aligned with the original WT sequence to determine mutations generated by the CRISPR-Cas9 treatment. Clones were designated as BV-2 AC7 biallelic KO clones, BV-2 AC7 monoallelic KO clones, and BV-2 WT clones.

### 4.9. Authentication of BV-2 Cell Clones

Two AC7 biallelic KO clones and one WT clone were chosen for further analyses. The five most likely off-target sites were selected using Cas-OFFinder [41]. The sequences of these potential off-target sites of the clones were isolated by PCR, determined, and compared with those of the parental BV-2 cells. The authenticity of the clones was examined by Species-specific PCR Evaluation and by short tandem repeat profiling (IDEXX Bioresearch, Columbia, MO, USA).

### 4.10. Phagocytosis Assay

BV-2 cells were transferred onto polyethyleneimine (PEI) coated 24-well plates (4 × 10^5^ cells/well) and adhered overnight. *E. coli* was labeled with Alexa Fluor 488 5-SDP Ester (Molecular Probes, Eugene, OR, USA), following the manufacturer’s protocol. Cells were treated with Alexa Fluor 488-labeled E. coli (MOI: 100) for one hour, harvested, and analyzed with flow cytometry (FACSCalibur, BD Biosciences, San Jose, CA, USA) for distribution and intensity of fluorescence signal of Alexa Fluor 488. The mean fluorescent intensity (MFI) was reported. Experiments were conducted in triplicate and repeated at least 3 times.

### 4.11. Bacterial Killing Assay

*E. coli* were cultured in LB medium at 225 rpm, 37 °C, one day before the experiment. BV-2 cells were transferred onto polyethyleneimine (PEI) coated 96-well plates (1 × 10^5^ cells/well) and adhered overnight. Cells were treated with *E. coli* (MOI: 1) for 20 min at 37 °C, then washed with fresh medium to remove extracellular bacteria. One set of wells was treated with 0.08% saponin to release intracellular bacteria. Another set of wells was incubated with fresh medium for 90 min before saponin treatment. Bacteria in each well were counted by colony formation on LB agar plates.

### 4.12. Oxidative Burst Assay

BV-2 cells were transferred onto polyethyleneimine (PEI) coated 24-well plates (4 × 10^5^ cells/well) and adhered overnight. Cells were pre-treated with 5 μg/mL DHR123 for 15 min, then treated with *E*. *coli* (MOI: 5) for 45 min. Cells were harvested and analyzed with flow cytometry for distribution and intensity of the green fluorescence signal of rhodamine 123. The MFI was reported. Experiments were conducted in triplicate and repeated at least 3 times.

### 4.13. Microglia Activation

BV-2 cells were activated into three different active states: classical activation (M1), alternative activation (M2a), and regulatory (M2b) as described previously [19]. M1 type of BV-2 cell was activated by 40 U/mL IFN-γ and 3 ng/mL Lipopolysaccharide (LPS) from *E. coli* O55: B5 for 24 h. M2a type of BV-2 cell was activated by 20 U/mL IL-4 for 8 h at 37 °C, 5% CO_2_. M2b type of BV-2 cell was activated by 10 μM PGE_1_ and 100 ng/mL LPS for 1 h at 37 °C, 5% CO_2_. BV-2 cell activation was characterized by the expression of inducible nitric oxide synthase (iNOS) for M1, arginase 1 (Arg1) for M2a, and IL-10 for M2b. In addition, M1 activation was assessed by the production of nitric oxide (NO).

### 4.14. RNA Isolation, Reverse Transcription, and Quantitative PCR

Cells were plated at a density of 3 × 10^6^ cells/well onto 6-well plates. Following activation as described above, cells were harvested using the Direct-zol RNA Miniprep kit (Zymo Research, Irvine, CA, USA) for total RNA extraction, in accordance with the manufacturer’s protocol. A High-Capacity cDNA Reverse Transcription Kit (Applied Biosystems, Foster City, CA, USA) was utilized to reverse transcribe 1000 ng of total RNA. Real-time PCR was performed by SYBR green-based detection system using an Applied Biosystems 7300 Real-Time PCR System and 7300 System software (Applied Biosystems, Foster City, CA, USA). Primers were designed to span exon-exon junctions using Primer-BLAST [45] and experimentally validated; PCR primers used are outlined in Table 2.

### 4.15. Nitric Oxide (NO) Production

The amount of nitrite in the culture medium was measured using Griess reagent as a substitute for NO production by BV-2 cells. BV-2 cells were transferred onto 96-well plates (1 × 10^5^ cells/well) and incubated for 2 h at 37 °C, 5% CO_2_. Cells were activated by 3 ng/mL LPS and up to 80 U/mL IFN-γ for 24 h at 37 °C, 5% CO_2_. 50 μL of cell culture medium was used for Griess Reagent Assay (Promega, Madison, WI, USA), as described in the manufacturer’s protocol. Experiments were conducted in triplicate and repeated at least 3 times.

### 4.16. Statistical Analysis

All values are reported as mean ± standard error of mean (SEM). Statistical significance of the differences (*p* < 0.05) was analyzed by one-way ANOVA or two-way repeated measures ANOVA. Pair-wise comparisons were carried out by Holm–Sidak method using SigmaStat v12.5 (SyStat Software, San Jose, CA, USA).

## Figures and Tables

**Table 1:** Base deletion/insertion in biallelic AC7 knockout (KO) BV-2 clones.

Clone		gRNA Target Sequence
	Deletion/Insertion	TCTCCACACGAAGCTTCCGACGG
1	−1 base	TCTCCACACGAAGCTT-CGACGG
2	−16 base	T----------------CGACGG
	−1 base	TCTCCACACGAAGCTT-CGACGG
3	−1 base	TCTCCACACGAAGCTT-CGACGG
4	−1 base	TCTCCACACGAAGCTT-CGACGG
	+1 base	TCTCCACACGAAGCTTCGCGACGG
5	−1 base	TCTCCACACGAAGCTT-CGACGG
6	−1 base	TCTCCACACGAAGCTT-CGACGG
	−2 base	TCTCCACACGAAGCT--CGACGG
7	−1 base	TCTCCACACGAAGCTT-CGACGG
8	−1 base	TCTCCACACGAAGCTT-CGACGG
9	−1 base	TCTCCACACGAAGCTT-CGACGG

**Table 2:** RT-qPCR primers.

	Forward (3′-5′)	Reverse (3′-5′)
IL-10	ATAACTGCACCCACTTCCCA	GGGCATCACTTCTACCAGGT
iNOS	CAGAGGACCCAGAGACAAGC	CAGCCTGGCCAGATGTTCCT
Arginase I	GGAATCTGCATGGGCAACCT	GGTCTACGTCTCGCAAGCCA
β-actin	CCTTCTACAATGAGCTGCGTGT	CTGGATGGCTACGTACATGGC

## Data Availability

Not applicable.
